# Underlying Geometric Flow in Hamiltonian Evolution

**DOI:** 10.3390/e27050510

**Published:** 2025-05-09

**Authors:** Gil Elgressy, Lawrence Horwitz

**Affiliations:** 1 Department of Physics, Bar Ilan University, Ramat Gan 52900, Israel; 2 Department of Physics, Ariel University, Ariel 44837, Israel; 3 School of Physics, Tel Aviv University, Ramat Aviv 69978, Israel

**Keywords:** Hamiltonian evolution, tensor metric operator, underlying geometric flow, adiabatic flow, metric operator space, quantum mechanical Ricci flow, *Hölder* spaces, convergence stability, local instability

## Abstract

In this paper, an underlying perturbed Ricci flow construction is made within the metric operator space, originating from the Heisenberg dynamical equations, to formulate a method which appears to provide a new geometric approach for the geometric formulation of the quantum mechanical dynamics. A quantum mechanical notion of stability and local instability is introduced within the quantum mechanical theory, based on the quantum mechanical dynamical equations governing the evolution of the tensor metric operator. The stability analysis is conducted in the topology of little $H\ddot{o}lder$ spaces of metrics which the tensor metric operator acts on. Finally, a theorem is introduced in an attempt to characterize the stability properties of the quantum mechanical system such that it brings the quantum mechanical dynamics into the analysis.

## 1. Introduction

We briefly introduce the work of Horwitz at al. [1], attempting to characterize chaotic Hamiltonian systems in terms of the curvature associated with a Riemannian metric tensor through definition of a conformal metric.

Given the classical Hamiltonian of form (1) in a curved space [1]

(1) $$H_{G}:=\frac{1}{2m}g_{ij}\left(x\right)p^{i}p^{j}$$

Following the Hamilton equations results in the geodesic equation

(2) $${\ddot{x}}_{l}=-\Gamma_{l}^{mn}{\dot{x}}_{m}{\dot{x}}_{n}$$

where $\Gamma_{l}^{mn}$ is the connection form.

Studying the separation of two nearby trajectories $\xi_{l}=x_{l}^{\prime }-x_{l}$ at *t*, which are the components of the geodesic deviation vector. Let γ be the parameter for a family of geodesics in the neighborhood of the coordinates $x_{l}\left(t\right)$ of a point on a geodesic defined by Equation (2) and $\xi_{l}\left(t\right)=\frac{\partial x_{l}\left(\gamma ,t\right)}{\partial \gamma }{|}_{\gamma =0}$.

Then, the second covariant derivative, D/Dt, of the geodesic deviation results in the following geodesic deviation equation [1]

(3) $$\frac{D^{2}\xi_{i}}{Dt^{2}}=R_{i}^{jlk}{\dot{x}}_{j}{\dot{x}}_{k}\xi_{l}$$

where $R_{i}^{jlk}$ are the components of the Riemann curvature tensor. The stability of the geodesic flow is locally determined by the geodesic deviation Equation (3) and therefore is locally characterized by the curvature of the manifold [1].

Next, to study the stability of the Hamiltonian evolution generated by

(4) $$H=\delta_{ij}\frac{p^{i}p^{j}}{2m}+V\left(y\right),$$

a geometric embedding of the Hamiltonian dynamics is constructed by the Hamiltonian $H_{G}$ in the form of Equation (1) with a conformal transformation of Equation (4), where the conformal factor results in [1]

(5) $$g_{ij}\left(x\right):=\Phi \left(x\right)\delta_{ij},\;\Phi \left(x\right):=\frac{E}{E-V(y)}\equiv F\left(y\right)$$

A relation is made with the set of coordinates $\left\{y^{j}\right\}$ of Equation (4) with the set of coordinates $\left\{x_{i}\right\}$ through the conformal factor. Two assumptions are made; 1. E is assumed to be the conserved value of *H* and $H_{G}$ 2. The momentum is the same for both of the dynamics governed by the respective Hamiltonians $H_{G}$ and *H* (curved and flat spaces, respectively).

Therefore, they are related by

(6) $$E-V\left(y\right)=\delta_{ij}\frac{p^{i}p^{j}}{2m}$$

Next, following Hamilton equations of both curved and flat spaces

(7) $$\begin{array}{c} {\dot{x}}_{i}=\frac{\partial H_{G}}{\partial p^{i}}=\frac{1}{m}g_{ij}p^{j}\\ {\dot{y}}^{i}:=\frac{1}{m}p^{i}=\frac{\partial H}{\partial p^{i}} \end{array}$$

and equating the momenta results in

(8) $$\begin{array}{c} {\dot{y}}^{j}=g^{ji}{\dot{x}}_{i} \end{array}$$

as a local tangent space transformation which reflects a geometric embedding of the original Hamiltonian motion. Clearly, this map is not integrable; so the global relation between {x} and {y} is not established by this map [1,2].

It follows from Equation (8) that

(9) $${\ddot{x}}_{l}=g_{lj}{\ddot{y}}^{j}+\frac{\partial g_{lj}}{\partial x_{n}}{\dot{x}}_{n}{\dot{y}}^{j}$$

Then, substituting Equation (9) in Equation (2) results in

(10) $${\ddot{y}}^{l}=-M_{mn}^{l}{\dot{y}}^{m}{\dot{y}}^{n},\;where\;M_{mn}^{l}:=\frac{1}{2}g^{lk}\frac{\partial g_{nm}}{\partial y^{k}}$$

which has the form of a geodesic equation with a reduced connection form that is completely covariant.

Following Horwitz, Yahalom et al. [3] prove that, by power series expansions, one may argue [1] that the two coordinate systems are involved with two coordinatizations. As a coordinate space, the $\left\{y^{l}\right\}$ are called the *Hamilton manifold* and $\left\{x_{l}\right\}$ are called the *Gutzwiller manifold* [1], with two different connection forms and are related locally by $\delta y^{j}:=g^{ji}\delta x_{i}$.

A curvature corresponding to the Hamilton manifold may be derived by the covariant derivative for a covariant tensor on the Gutzwiller manifold [1] with the connection form, to derive a covariant derivative in the Hamilton manifold (for the formula for curvature)

(11) $$A^{m;q}=\frac{\partial A^{m}}{\partial x_{q}}-\Gamma_{k}^{mq}A^{k}$$

and induced connection form (lowering the index q with $g_{lq}$),

(12) $$\Gamma_{lk}^{m}\equiv g_{lq}\Gamma_{k}^{mq}=\frac{1}{2}g^{mq}\left(\frac{\partial g_{lq}}{\partial y^{k}}-\frac{\partial g_{kq}}{\partial y^{l}}-\frac{\partial g_{kl}}{\partial y^{q}}\right)$$

Since it is antisymmetric in its lower indices (l, k) (implying the existence of torsion), along geodesic motion, it results in the symmetric connection form (10) [1].

These derived expressions for the geodesic equation and connection form corresponding to the Hamilton manifold (not metric-compatible connections) may be explained by a parallel transport on the local flat tangent space of the Gutzwiller manifold (whose tensor metric is $g_{ij}$) which results in the “truncated” connection (10) [1] if a transformation is made to reach the Hamilton manifold (by raising the tensor index).

Next, to compute the geodesic deviation in the Hamilton manifold, one can construct a covariant derivative such that the second-order geodesic deviation equations result in [1]

(13) $$\frac{D^{2}\xi^{l}}{Dt^{2}}=R_{qmn}^{l}{\dot{y}}^{q}{\dot{y}}^{n}\xi^{m}$$

where

(14) $$R_{qmn}^{l}=\frac{\partial M_{qm}^{l}}{\partial y^{n}}-\frac{\partial M_{qn}^{l}}{\partial y^{m}}+M_{qm}^{k}M_{nk}^{l}-M_{qn}^{k}M_{mk}^{l}$$

to be called the *dynamical curvature* [1].

Horwitz et al. conjectured that this structure of Equation (13) can be used as a local criterion to characterize the stability of the original Hamiltonian motion [1,4], picturing local regions of instability which are conjectured to drive the motion into a chaotic behavior. Instability in the classical case occurs if at least one of the eigenvalues of the dynamical curvature is negative [1,4].

In a previous work [5], we suggested extending these ideas to a quantum mechanical framework. We studied the quantum theory associated with a general operator-valued Hermitian Riemannian Hamiltonian

(15) $${\hat{H}}_{G}:=\frac{1}{2m}p^{i}g_{ij}\left(x\right)p^{j}$$

with canonical commutation relations

(16) $$\left[x_{i},p^{j}\right]=i\hbar \delta_{i}^{j}$$

Following the Heisenberg picture results in

(17) $${\dot{x}}_{k}=\frac{1}{2m}\left\{p^{i},g_{ik}\right\}$$

and

(18) $$p^{l}=\frac{m}{2}\left\{{\dot{x}}_{k},g^{kl}\right\}$$

We derived from the Heisenberg equations the quantum mechanical form of the “geodesic” equation for ${\ddot{x}}_{l}$ generated by the Hamiltonian ${\hat{H}}_{G}$,

(19) $${\ddot{x}}_{l}=\frac{1}{16}\left(\left\{\left\{\left\{g^{nm},{\dot{x}}_{m}\right\},\frac{\partial g_{ln}}{\partial x_{i}}\right\},g_{ij}\left\{g^{jp},{\dot{x}}_{p}\right\}\right\}-2\left\{g^{ip},{\dot{x}}_{p}\right\}g_{ln}\frac{\partial g_{ij}}{\partial x_{n}}\left\{g^{jp},{\dot{x}}_{q}\right\}\right)$$

where the classical limit of Equation (19) results in the same conventional structure of a geodesic flow generated by a classical Hamiltonian of the form (1) [1]

(20) $${\ddot{x}}_{l}=-\Gamma_{l}^{pq}{\dot{x}}_{p}{\dot{x}}_{q}$$

with

(21) $$\Gamma_{l}^{pq}=\frac{1}{2}g_{ln}\left(\frac{\partial g^{nq}}{\partial x_{p}}+\frac{\partial g^{np}}{\partial x_{q}}-\frac{\partial g^{pq}}{\partial x_{n}}\right)$$

In analogy to the classical case, quantization of Equation (8) yields a new set of operators

(22) $${\dot{y}}^{l}:=\frac{1}{2}\left\{{\dot{x}}_{k},g^{kl}\right\}$$

Substituting Equation (18) in Equation (22) results in the structure analogous to the classical form

(23) $$p^{l}=m{\dot{y}}^{l}$$

Following the Heisenberg picture, the second-order equation for the dynamical variable {y}

(24) $${\ddot{y}}^{l}=-\frac{1}{2}{\dot{y}}^{i}\frac{\partial g_{ij}}{\partial x_{l}}{\dot{y}}^{j}$$

closely related to the form obtained in the classical case for the “geodesic” equation (Equation (10)) with reduced connection [1].

Moreover, the quantum “geodesic” formula Equation (19) reflects a property of the quantum mechanical nature. This can be seen by expressing it explicitly in terms of the canonical momenta using (22) and (23), to get a form of a bilinear in momentum ordered to bring momenta to the left and right, to obtain

(25) $$\begin{array}{c} {\ddot{x}}_{l}=\frac{1}{2m^{2}}p^{i}\left(\frac{\partial g_{li}}{\partial x_{n}}g_{nj}+\frac{\partial g_{lj}}{\partial x_{n}}g_{ni}-\frac{\partial g_{ij}}{\partial x_{n}}g_{ln}\right)p^{j}+\frac{1}{4m^{2}}\frac{\partial }{\partial x_{j}}\left(\frac{\partial^{2}g_{ln}}{\partial x_{i}\partial x_{n}}g_{ij}\right) \end{array}$$

where the first term is closely related to the classical connection form, and the second term is an essentially quantum effect. This property is consistently manifested, originated by the natural property of quantum mechanics, as it was also observed in our latest work [6].

In the $\left\{y^{j}\right\}$ set of coordinates, it follows from [5] that the commutation relation between the momenta and the coordinate operators $\left\{y^{j}\right\}$ is

(26) $$\left[p^{n},y^{l}\right]=-i\hbar g^{nl}\left(x\right)$$

Then, formally, a transformation between the two coordinate bases, $\left\{x_{i}\right\}$ and $\left\{y^{j}\right\}$, is made to express $\delta \left(\psi_{t},{\ddot{y}}^{l}\psi_{t}\right)\left(t\right)$ in the {y} coordinate system, where, for “geodesic deviation” [5,7,8], we suggested inducing a deviation in the Ehrenfest approximation to the trajectory, for a smooth function $\psi_{t}\left(x\right)$, by the generator of translation such that $\psi_{t}\left(x\right)\to \psi_{t}\left(x+\xi \right)$. A “deviation operator” is introduced to define “local instability” in the quantum theory.

We simulated several quantum dynamical systems and investigated the behavior of the orbits of expectation values of {y}. We demonstrated the correlation between the complexity and properties of the orbits pictured by the expectation values and the predictions of local instability corresponding to deviation under small perturbation, in corresponding with the classical problem [5,9,10,11,12,13,14].

In this work, we attempt to address the basic problem of quantum chaos. We provide a geometric structure within the operator space in the Heisenberg picture with detailed analysis based on the geometric flow properties within the operator space. A theorem is made that relates the stability properties of the geometric flow within the metric operator space with the stability properties of the quantum mechanical dynamics. A formal definition is suggested for “local instability” in the quantum theory.

## 2. Emergence of an Underlying Geometric Flow in the Quantum Theory

We seek to develop a method that incorporates quantum mechanical dynamics into the analysis with the dependency on initial conditions rather then the dynamical equations that govern the evolution of the trajectories defined by the expectation values.

### 2.1. Representation Theory in the {x} Coordinates

In the present work, we introduce a new approach which concerns the problem of geometrizing the quantum mechanical dynamics involved in the evolution of the tensor metric operator $g_{ij}\left(x\right)$ in the Heisenberg picture, evolved in time by a geometric flow which is essentially originated from the evolution generated by the geometric Hamiltonian operator in a form defined below.

**Definition 1.**  *(Geometric Hamiltonian operator)*

*Let the position and momentum operators x and p satisfy the canonical commutation relation*

(27) $$\begin{array}{c} xp-px=i\hbar I \end{array}$$

*and*

(28) $$\left[x_{i},x_{j}\right]=\left[p^{i},p^{j}\right]=0$$

*Let $H_{G}\left(R^{n}\right):=L^{2}\left(R^{n}\right)$ be a Hilbert space corresponding to a given quantum mechanical system and let the tensor metric operator $g_{ij}\left(x\right)$ be a Hermitian function of the position operator x acting on $H_{G}$ where x and p are as above.*

*We define the geometric Hamiltonian operator-valued Hermitian Riemannian Hamiltonian to be*

(29) $$\begin{array}{c} {\hat{H}}_{G}:=\frac{1}{2m}p^{i}g_{ij}\left(x\right)p^{j} \end{array}$$

*where ${\hat{H}}_{G}$ is a self-adjoint geometric Hamiltonian operator generating the evolution of the system.*

In this work, we also achieve a quantum mechanical embedding of the geometrical structure where we follow some basic ideas introduced by us in previous works [5,6].

We start first by distinguishing the Schrodinger picture (notated by *S*) from Heisenberg operators (notated by *H*):

(30) $$\begin{array}{c} g_{ij}\left(t\right)=\langle \psi \left(t\right)|g_{ij}{|\psi \left(t\right)\rangle }_{S}=\langle \psi \left(t_{0}\right)|U^{†}g_{ij}U|\psi \left(t_{0}\right)\rangle_{S}=\langle \psi |g_{ij}\left(t\right){|\psi \rangle }_{H} \end{array}$$

where the operator is defined as

(31) $$\begin{array}{c} {g_{ij}}_{H}\left(t\right)=U^{†}\left(t,t_{0}\right){g_{ij}}_{S}U\left(t,t_{0}\right)\\ {g_{ij}}_{H}\left(t_{0}\right)={g_{ij}}_{S} \end{array}$$

The time-evolution of the operators following the Heisenberg algebra with the geometric Hamiltonian operator in the form of Equation (29) is:

(32) $$\begin{array}{c} \frac{\partial {g_{ij}}_{H}}{\partial t}=\frac{\partial }{\partial t}\left(U^{†}{g_{ij}}_{S}U\right)=\frac{\partial U^{†}}{\partial t}{g_{ij}}_{S}U+U^{†}{g_{ij}}_{S}\frac{\partial U}{\partial t}+U^{†}\frac{\partial {g_{ij}}_{S}}{\partial t}U\\ =\frac{i}{\hbar }U^{†}{\hat{H}}_{G}{g_{ij}}_{S}U-\frac{i}{\hbar }U^{†}{g_{ij}}_{S}{\hat{H}}_{G}U+{\left(\frac{\partial g_{ij}}{\partial t}\right)}_{H}\\ =\frac{i}{\hbar }H_{H}{g_{ij}}_{H}-\frac{i}{\hbar }{g_{ij}}_{H}H_{H}=-\frac{i}{\hbar }{\left[g_{ij},{\hat{H}}_{G}\right]}_{H} \end{array}$$

where $H_{H}=U^{†}{\hat{H}}_{G}U$, that results in

(33) $$\begin{array}{c} \frac{\partial {g_{ij}}_{H}}{\partial t}=-\frac{i}{\hbar }{\left[g_{ij},{\hat{H}}_{G}\right]}_{H} \end{array}$$

and is known as the Heisenberg equation of motion.

Since ${\hat{H}}_{G}$ is a time-independent Hamiltonian, $H_{H}={\hat{H}}_{G}$, and the Heisenberg equation results in

(34) $$\begin{array}{c} \frac{\partial {g_{ij}}_{H}}{\partial t}=-\frac{i}{\hbar }\left[{g_{ij}}_{H},{\hat{H}}_{G}\right] \end{array}$$

For simplicity, we drop the notation ${g_{ij}}_{H}$ and write $g_{ij}$ in the rest of our present work.

Next, we explicitly calculate the time evolution of the tensor metric operator $g_{ij}\left(x\right)$ in the Heisenberg picture following Equation (34), which results in

(35) $$\begin{array}{c} \frac{\partial g_{ij}}{\partial t}=\frac{i}{\hbar }\left[{\hat{H}}_{G},g_{ij}\right]=\frac{i}{2m\hbar }\left[p^{n}g_{nm}p^{m},g_{ij}\right]=\\ \frac{i}{2m\hbar }\left(\left[p^{n},g_{ij}\right]g_{nm}p^{m}+p^{n}g_{nm}\left[p^{m},g_{ij}\right]\right)=\frac{1}{2m}\left(\frac{\partial g_{ij}}{\partial x_{n}}g_{nm}p^{m}+p^{n}g_{nm}\frac{\partial g_{ij}}{\partial x_{m}}\right) \end{array}$$

such that

(36) $$\begin{array}{c} \frac{\partial g_{ij}}{\partial t}=\frac{1}{m}\left(M_{ij}^{n}g_{nm}p^{m}+p^{n}g_{nm}M_{ij}^{m}\right)=\frac{1}{m}\left\{p^{n},g_{nm}M_{ij}^{m}\right\}\\ \mathrm{where}\;M_{ij}^{n}:=\frac{1}{2}\frac{\partial g_{ij}}{\partial x_{n}} \end{array}$$

Next, expressing Equation (36) explicitly, substituting anticommutator identities satisfies the following expression

(37) $$\begin{array}{c} \frac{\partial g_{ij}}{\partial t}=\frac{1}{m}\left\{p^{n},g_{nm}M_{ij}^{m}\right\}=\frac{1}{m}\left[p^{n},g_{nm}M_{ij}^{m}\right]+\frac{2}{m}g_{nm}M_{ij}^{m}p^{n}\\ =\frac{-i\hbar }{m}\frac{\partial (g_{nm}M_{ij}^{m})}{\partial x_{n}}+\frac{2}{m}g_{nm}M_{ij}^{m}p^{n}\\ =\frac{-2i\hbar }{m}M_{nm}^{n}M_{ij}^{m}+\frac{-i\hbar }{m}g_{nm}\frac{\partial M_{ij}^{m}}{\partial x_{n}}+\frac{2}{m}g_{nm}M_{ij}^{m}p^{n} \end{array}$$

### 2.2. Representation Theory in the {y} Coordinates

Following our previous works [5,6], we now define a new set of Hermitian operators $\left\{y_{H}^{l}\left(x\right)\right\}$ such that the commutation relations between the momenta and the operators $\left\{y^{j}\right\}$ are given by a form as follows, in correspondence with Equation (26), given in our previous work [5],

(38) $${\left[p^{n},y^{l}\right]}_{H}:=-2i\hbar g_{H}^{nl}\left(x\right)$$

with a tensor metric operator in a general operator-valued Hermitian form.

The canonical commutation relations, Equation (27), applied to the left side of Equation (38) results in

(39) $${\left[p^{n},y^{l}\right]}_{H}=-i\hbar \frac{\partial y^{l}}{\partial x_{n}}=-2i\hbar g^{nl}\left(x,t\right)$$

Therefore, one may define the following relation (for a full discussion of this point see [5])

(40) $$\frac{\partial g_{ij}}{\partial x_{l}}:=2g^{lm}\frac{\partial g_{ij}}{\partial y^{m}}$$

as a local relation between the two sets of coordinates, suggesting that, as a result of such a definition, one may think formally of a transformation between the two coordinate bases, $\left\{x_{i}\right\}$ and $\left\{y^{j}\right\}$ [5,6].

Applying the Heisenberg picture for the variables corresponding to $\left\{y^{l}\right\}$, and substituting Equation (38), results in

(41) $$\begin{array}{c} {\dot{y}}^{l}=\frac{i}{\hbar }\left[{\hat{H}}_{G},y^{l}\right]=\frac{i}{2m\hbar }\left[p^{i}g_{ij}p^{j},y^{l}\right]=\\ \frac{i}{2m\hbar }\left(\left[p^{i},y^{l}\right]g_{ij}p^{j}+p^{i}\left[g_{ij},y^{l}\right]p^{j}+p^{i}g_{ij}\left[p^{j},y^{l}\right]\right) \end{array}$$

such that it satisfies the following relation with the momentum operator

(42) $$\begin{array}{c} {\dot{y}}^{l}=\frac{2}{m}p^{l}=\left\{{\dot{x}}_{k},g^{kl}\right\} \end{array}$$

substituting Equation (18), which is closely related to the form given in definition Equation (22) [5].

In analogy to our previous work [6] and DeWitt’s work [15] introducing DeWitt’s point transformation formula for the quantum transformation law for the momentum operators [15], to designate general point transformations between the momentum operator *p* expressed in the {x} space representation and the $p^{\prime }$ expressed in the {y} space representation, covariant under point transformations, we define

(43) $$\begin{array}{c} p_{l}^{\prime }=\frac{\partial x_{i}}{\partial y^{l}}p^{i}+\frac{1}{2}\left[p^{i},\frac{\partial x_{i}}{\partial y^{l}}\right]=\frac{1}{2}\left\{p^{i},\frac{\partial x_{i}}{\partial y^{l}}\right\} \end{array}$$

Substituting Equation (39)–(40), a local relation between the two sets of coordinates $\left\{x_{j}\right\}$ and $\left\{y^{i}\right\}$, the momentum operator $p_{l}^{\prime }$ becomes

(44) $$p_{l}^{\prime }=-i\hbar \frac{\partial }{\partial y^{l}}-\frac{1}{2}i\hbar M_{li}^{i},\;\mathrm{where}\;M_{li}^{i}:=g^{in}\frac{\partial g_{li}}{\partial y^{n}}$$

so that $M_{li}^{i}$ has the same form (up to a factor of two) as the reduced connection form in the classical case (Equation (10)) and is consistent with Equation (36), correctly symmetrized so as to make it Hermitian.

Note that $p_{l}^{\prime }\left(t\right)$ is time-dependent via the dependency of $M_{li}^{i}$ on g(x,t) which gives the origin for the underlying geometry (M,g(x,t)) involved with the {y} space representation.

Furthermore, Equation (17) is substituted so that Equation (43) for the momentum operator $p_{l}^{\prime }$ implies the following relation

(45) $$\begin{array}{c} p_{l}^{\prime }=\frac{1}{4}\left\{g_{li},p^{i}\right\}=\frac{m}{2}{\dot{x}}_{l} \end{array}$$

The relations between the two momentum operators, substituting $p^{i}\to -i\hbar \frac{\partial }{\partial x_{i}}$ in Equation (43), are

(46) $$p_{l}^{\prime }=\frac{1}{2}g_{li}p^{i}-\frac{1}{2}i\hbar M_{li}^{i}$$

Then, the commutation relations result in

(47) $$\begin{array}{c} {}[p^{n},x_{l}]=-i\hbar \delta_{l}^{n}\\ {}[p^{n},y^{l}]=-2i\hbar g^{nl}\\ {}[p_{n}^{\prime },x_{l}]=-\frac{1}{2}i\hbar g_{nl}\\ {}[p_{n}^{\prime },y^{l}]=-i\hbar \delta_{n}^{l} \end{array}$$

The relations between the momenta and the velocities $\left\{{\dot{y}}^{n}\right\}$ are (from Equations (45) and (42)), so that

(48) $$\begin{array}{c} p^{l}=\frac{m}{2}{\dot{y}}^{l}=\left\{\frac{m}{2}{\dot{x}}_{k},g^{kl}\right\}\\ p_{l}^{\prime }=\frac{1}{8}m\left\{{\dot{y}}^{n},g_{ln}\right\}=\frac{1}{4}\left\{p^{n},g_{ln}\right\}=\frac{m}{2}{\dot{x}}_{l} \end{array}$$

is consistent with Equations (17) and (18) and closely related with the definition in Equations (22) and (23) [5], along with the definition for $p_{l}^{\prime }$ (Equation (43)).

Therefore, in analogy to our previous work [6] where we worked with two space representations, motivated by the work of Horwitz et al. [1] on the stability of classical Hamiltonian systems by geometrical methods, where a coordinate space, $\left\{y^{l}\right\}$, which is called the *Hamilton manifold*, is endowed with a connection form $M_{mn}^{l}$ (Equation (10)) and what is called the dynamical curvature. It is not uniquely defined in terms of the original manifold $\left\{x_{l}\right\}$, which is called the *Gutzwiller manifold*.

To rigorously complete our mathematical formulation, we define two space representations, in analogy to our previous work [6], corresponding to the {x} space representation and the {y} space representation:

**Definition 2.**  *(Gutzwiller representation)*

*Let $H_{G}$ be a Hilbert space corresponding to a given quantum mechanical system, and let ${\hat{H}}_{G}$ be the self-adjoint geometric Hamiltonian generating the evolution of the system as before. Let the position and momentum operators, x and p, be as before and satisfy the canonical commutation relations (CCR).*

*The position space wave functions, expressed in the {x} space representation*

(49) $$\psi \left(x,t\right)=\int_{R}\psi \left(x^{\prime },t\right)\langle x|x^{\prime }\rangle dx^{\prime }$$

*where $\psi \left(x,t\right)\in L^{2}\left(R,dx\right)$, are then said to be the Gutzwiller representation.*

**Definition 3.**  *(Hamilton representation)*

*Let $H_{G}$ be a Hilbert space corresponding to a given quantum mechanical system, and let ${\hat{H}}_{G}$ be the self-adjoint geometric Hamiltonian generating the evolution of the system as before.*

*Let the position and momentum operators, x and p, be as before, and let the position and momentum operators, y and $p^{\prime }$, be as before*

(50) $$\begin{array}{c} {p^{\prime }}_{l}\left(t\right):=-i\hbar \frac{\partial }{\partial y^{l}}-\frac{1}{2}i\hbar M_{li}^{i},\;\mathrm{where}\;M_{li}^{i}\left(t\right):=g^{in}\frac{\partial g_{li}}{\partial y^{n}} \end{array}$$

*and satisfy the commutation relations*

(51) $$[p_{n}^{\prime },y^{l}{]}_{H}=-i\hbar \delta_{n}^{l}$$

*The position space wavefunctions are said to be the Hamilton representation, when expressed in the {y} space representation*

(52) $$\tilde{\varphi }\left(y,t\right):=\langle y|\varphi \rangle \left(t\right)=\int_{M}\varphi \left(y^{\prime },t\right)\langle y|y^{\prime }\rangle d\omega^{\prime }$$

*where $d\omega^{\prime }$ denotes the volume element, and the integration is to be carried out over the entire range of coordinate values $\tilde{\varphi }\left(y,t\right)\in L^{2}$.*

Note that the Hamilton representation carries an integration over the underlying manifold (M,g(x,t)) which is induced by the tensor metric operator $g_{H}\left(x,t\right)$.

### 2.3. Local Existence of an Underlying Ricci Flow in the Quantum Theory

In this section, encouraged to find a method which brings the quantum mechanical dynamics into the analysis of quantum chaos, we then construct a generalized form of the dynamical equations in the Heisenberg picture governing the evolution of the tensor metric operator $g_{ij}\left(x\right)$, Equation (37), in the form of a “geometric flow” [16,17,18].

Our so-obtained construction carries a structure closely related in form to the classical Ricci flow equations, which are time-evolution equations, i.e., equations of motion equal to the first-order time derivatives of $g_{ij}$.

We start by following our previous analysis of the Heisenberg picture for the tensor metric operator $g_{ij}\left(x\right)$ as a Heisenberg dynamical variable (Equation (37)), satisfying the Heisenberg’s form for the equations of motion expressed in the {x} representation. Following our previous formal definition for a transformation between the two space representations $\left\{x_{i}\right\}$ and $\left\{y^{j}\right\}$, Equation (37) expressed in the {y} space representation results in

(53) $$\begin{array}{c} \frac{\partial g_{ij}}{\partial t}=\frac{-2i\hbar }{m}M_{nm}^{n}M_{ij}^{m}+\frac{-i\hbar }{m}g_{nm}\frac{\partial M_{ij}^{m}}{\partial x_{n}}+\frac{2}{m}g_{nm}M_{ij}^{m}p^{n}\\ =\frac{-2i\hbar }{m}M_{nm}^{n}M_{ij}^{m}+\frac{-2i\hbar }{m}\frac{\partial M_{ij}^{m}}{\partial y^{m}}+\frac{2}{m}g_{nm}M_{ij}^{m}p_{n}^{\prime } \end{array}$$

Note that the momentum is expressed in the {y} space representation, covariant under point transformations between the two space representations [6].

Then, we express the momentum operator $p^{\prime }$ by the substitution of Equation (48) to relate the momentum $p^{\prime }$ with the velocity field expressed in the {y} space representation. A relation between the two dynamical evolutions of the operators $g_{H}$ and $y_{H}$ is obtained,

(54) $$\begin{array}{c} \frac{\partial g_{ij}}{\partial t}=\frac{-2i\hbar }{m}M_{nm}^{n}M_{ij}^{m}+\frac{-2i\hbar }{m}\frac{\partial M_{ij}^{m}}{\partial y^{m}}+\frac{1}{4}g_{nm}M_{ij}^{m}\left\{{\dot{y}}^{k},g_{nk}\right\} \end{array}$$

Next, we define the “*Riemann curvature*” operator in the formal form

(55) $$R_{qmn}^{l}:=\frac{\partial M_{qm}^{l}}{\partial y^{n}}-\frac{\partial M_{qn}^{l}}{\partial y^{m}}+M_{qm}^{k}M_{nk}^{l}-M_{qn}^{k}M_{mk}^{l}$$

expressed in the {y} space representation, to be a Hermitian function of the position operator *x* (since $g_{ij}\left(x\right)$ is a c-number-valued function of the *x* coordinate base) acting on $H_{G}$, equivalent to the classical form of the Riemann curvature tensor in the $\left\{y^{l}\right\}$ coordinate base (associated with the geodesic deviation equation in the Hamilton manifold, accounting for the dynamical curvature in Equation (14) [1]).

We define, as well, the “*Ricci curvature*” operator in a formal form to be

(56) $$R_{ij}:=R_{ilj}^{l}$$

classically equivalent in form to the Ricci curvature tensor obtained by taking the contraction of the second and fourth indices of the Riemann curvature tensor.

We suggest, as an analogy to the classical case, a formal construction for a quantum mechanical “Ricci flow” in a generalized form, associated with the general operator-valued Hermitian geometric Hamiltonian of the form (29) following the Heisenberg algebra with the geometric Hamiltonian. The quantum mechanical “Ricci flow” equation is expressed by the representation theory in the {y} coordinates on the Hilbert space $H_{G}$ corresponding to a given quantum mechanical system.

Substituting Equations (55) and (56) in Equation (53) results in

(57) $$\begin{array}{c} \frac{\partial g_{ij}}{\partial t}=\frac{2i\hbar }{m}R_{ij}+\frac{-2i\hbar }{m}\left(\frac{\partial M_{ai}^{a}}{\partial y^{j}}+M_{ib}^{a}M_{aj}^{b}\right)+\frac{2}{m}g_{nm}M_{ij}^{m}p_{n}^{\prime }\\ =\frac{2i\hbar }{m}R_{ij}+\frac{-2i\hbar }{m}\left(\frac{\partial M_{ai}^{a}}{\partial y^{j}}+M_{ib}^{a}M_{aj}^{b}-\frac{-i}{\hbar }g_{nm}M_{ij}^{m}p_{n}^{\prime }\right) \end{array}$$

where $R_{ij}$ is the Ricci operator defined in Equation (56).

Next, given the structure of Equation (57), we define a new operator in a generalized form to be an additional term to the Ricci operator in the quantum mechanical dynamical Equation (57), formally defined as follows

(58) $$\begin{array}{c} \Xi_{ij}:=\frac{1}{2\lambda }\frac{\partial g_{ij}}{\partial t}+R_{ij},\;where\;\lambda :=\frac{i\hbar }{m} \end{array}$$

and $\Xi_{ij}$ is a general symmetric operator acting on $H_{G}$.

In what follows, motivated by the structure implied by Equation (57), we focus on the topic of geometrization of the quantum mechanical dynamics in the study of geometric flows. We discuss only Ricci flow here but analogues of these results may apply more broadly.

First, we define the *adiabatic flow*, then, the tensor metric operator $g_{ij}$ is used to define a “distance”, to be described below, within the operator space, so that it becomes a metric space.

The general form of $\Xi_{ij}$ associated with the evolution generated by the geometric Hamiltonian in the form of Equation (29), following Equation (57), is

(59) $$\Xi_{ij}=\frac{\partial M_{ai}^{a}}{\partial y^{j}}+M_{ib}^{a}M_{aj}^{b}-\frac{-i}{\hbar }g_{nm}M_{ij}^{m}p_{n}^{\prime }$$

such that, expressed in the {y} space representation (substitution of Equation (48))

(60) $$\Xi_{ij}=\frac{\partial M_{ai}^{a}}{\partial y^{j}}+M_{ib}^{a}M_{aj}^{b}-\frac{-im}{8\hbar }g_{nm}M_{ij}^{m}\left\{{\dot{y}}^{k},g_{nk}\right\}$$

is obtained. Next, we define an adiabatic quantum mechanical evolution. We start by following operator-valued analysis of the $\Xi_{ij}$ operator Equation (59) which results in

(61) $$\begin{array}{c} \Xi_{ij}g^{nl}+g^{nl}\Xi_{ij}=\left(\frac{\partial M_{ai}^{a}}{\partial y^{j}}+M_{ib}^{a}M_{aj}^{b}\right)g^{nl}+g^{nl}\left(\frac{\partial M_{ai}^{a}}{\partial y^{j}}+M_{ib}^{a}M_{aj}^{b}\right)-\frac{-i}{\hbar }\left(g_{nm}M_{ij}^{m}p_{n}^{\prime }g^{nl}+g^{nl}g_{nm}M_{ij}^{m}p_{n}^{\prime }\right)\\ \frac{\hbar }{-i}\left\{\Xi_{ij},g^{nl}\right\}=\frac{\hbar }{-i}\left\{\left(\frac{\partial M_{ai}^{a}}{\partial y^{j}}+M_{ib}^{a}M_{aj}^{b}\right),g^{nl}\right\}-\left(g_{nm}M_{ij}^{m}\right)\left(p_{n}^{\prime }g^{nl}+g^{nl}p_{n}^{\prime }\right)\\ \frac{\hbar }{-i}\left(g^{nm}M_{m}^{ij}\right)\left\{\Xi_{ij},g^{nl}\right\}=\frac{\hbar }{-i}\left(g^{nm}M_{m}^{ij}\right)\left\{\left(\frac{\partial M_{ai}^{a}}{\partial y^{j}}+M_{ib}^{a}M_{aj}^{b}\right),g^{nl}\right\}-\left(p_{n}^{\prime }g^{nl}+g^{nl}p_{n}^{\prime }\right) \end{array}$$

where we assume that $M_{ij}^{n}$ is invertible, so that $M_{m}^{ij}M_{ij}^{m}=M_{ij}^{m}M_{m}^{ij}=I_{n}$, while $g^{ij}g_{ij}=g_{ij}g^{ij}=I_{n}$ and $I_{n}$ denotes the n-by-n identity matrix.

Substituting Equation (48), i.e., ${\dot{y}}^{k}=\frac{2}{m}\left\{p_{i}^{\prime },g^{ik}\right\}$, in Equation (61) satisfies the following relation

(62) $$\begin{array}{c} \frac{2\hbar }{-mi}\left(g^{nm}M_{m}^{ij}\right)\left\{\Xi_{ij},g^{nl}\right\}=\frac{2i\hbar }{m}\left(g^{nm}M_{m}^{ij}\right)\left\{\left(\frac{\partial M_{ai}^{a}}{\partial y^{j}}+M_{ib}^{a}M_{aj}^{b}\right),g^{nl}\right\}-{\dot{y}}^{l} \end{array}$$

Define $A^{l}$ to be a general operator acting on $H_{G}$, giving Equation (58), such that

(63) $$\begin{array}{c} A^{l}=\frac{2\hbar }{-mi}\left(g^{nm}M_{m}^{ij}\right)\left\{\Xi_{ij},g^{nl}\right\} \end{array}$$

In the special case of Equation (59), the $A^{l}$ operator, yielding Equation (62), results in

(64) $$A^{l}=\frac{2i\hbar }{m}\left(g^{nm}M_{m}^{ij}\right)\left\{\left(\frac{\partial M_{ai}^{a}}{\partial y^{j}}+M_{ib}^{a}M_{aj}^{b}\right),g^{nl}\right\}-{\dot{y}}^{l}$$

We now consider the case of a bounded $A^{l}$ operator, considered to be small, in a manner which ensures that

(65) $${\dot{y}}^{l}\approx \frac{2\hbar }{m}\left(g^{nm}M_{m}^{ij}\right)\left\{\left(\frac{\partial M_{ai}^{a}}{\partial y^{j}}+M_{ib}^{a}M_{aj}^{b}\right),g^{nl}\right\}$$

Equation (65) is in a structure that bounds the momentum *p* in the case that the right-hand side of Equation (65) is bounded (i.e., manifolds with bounded geometry, to be described below).

Substituting Equation (65) in Equation (60) results in

(66) $$\begin{array}{c} \Xi_{ij}=\frac{\partial M_{ai}^{a}}{\partial y^{j}}+M_{ib}^{a}M_{aj}^{b}-\frac{-im}{8\hbar }g_{nm}M_{ij}^{m}\left\{{\dot{y}}^{k},g_{nk}\right\}\\ \approx \frac{m}{4\hbar }g_{ml}M_{ij}^{m}{\dot{y}}^{l}-\frac{-im}{8\hbar }g_{nm}M_{ij}^{m}\left\{{\dot{y}}^{k},g_{nk}\right\}\\ =\frac{-im}{4\hbar }\left(ig_{ml}M_{ij}^{m}{\dot{y}}^{l}-\frac{1}{2}g_{nm}M_{ij}^{m}\left\{{\dot{y}}^{k},g_{nk}\right\}\right)\\ =\frac{-im}{4\hbar }\left(ig_{ml}M_{ij}^{m}{\dot{y}}^{l}-\frac{1}{2}g_{nm}M_{ij}^{m}\left[{\dot{y}}^{k},g_{nk}\right]-g_{nm}M_{ij}^{m}g_{nk}{\dot{y}}^{k}\right) \end{array}$$

which ensures that $\Xi_{ij}$ is small as well.

Further operator-valued analysis results in

(67) $$\begin{array}{c} \Xi_{ij}\approx \frac{im}{8\hbar }g_{nm}M_{ij}^{m}\left[{\dot{y}}^{k},g_{nk}\right]+\frac{-im}{4\hbar }\left(ig_{ml}M_{ij}^{m}{\dot{y}}^{l}-g_{nm}M_{ij}^{m}g_{nk}{\dot{y}}^{k}\right)\\ \Xi_{ij}\approx \frac{im}{8\hbar }g_{nm}M_{ij}^{m}\left[\frac{i}{\hbar }\left[{\hat{H}}_{G},y^{k}\right],g_{nk}\right]+\frac{-im}{4\hbar }\left(ig_{ml}M_{ij}^{m}{\dot{y}}^{l}-g_{nm}M_{ij}^{m}g_{nk}{\dot{y}}^{k}\right)\\ =\frac{im}{8\hbar }g_{nm}M_{ij}^{m}\left[\frac{\partial g_{nk}}{\partial t},y^{k}\right]+\frac{-im}{4\hbar }\left(ig_{ml}M_{ij}^{m}{\dot{y}}^{l}-g_{nm}M_{ij}^{m}g_{nk}{\dot{y}}^{k}\right) \end{array}$$

implying that the tensor metric operator is *adiabatically* changing in a sense that the commutation relation of the rate of change of the tensor metric operator with the position operator is small and ${\dot{y}}^{n}\approx \frac{1}{2}\left[\frac{\partial g_{nk}}{\partial t},y^{k}\right]$.

In our recent work on the subject of quantum mechanical Hamiltonian Evolution on a Finsler Manifold, generated by the self-adjoint geometric Hamiltonian ${\hat{H}}_{G}=\frac{1}{2m}p^{i}g_{ij}\left(x,p\right)p^{j}$ [6], we found results with dynamical equations governing the evolution of the trajectories defined by the expectation values of the position expressed in the {y(x,p)} space representation in a structure of two terms, where the first term is the quantum mechanical form of a “geodesic flow”, with a connection form as in Equation (44) (up to a factor of two), and with a second term which is an essentially quantum effect. Note that this term, which was derived from the adiabatic case of a slowly changing momentum operator in a Finsler geometry, is in the form of $\frac{-m}{2}\frac{d}{dt}\left[i\hbar g_{ij},y^{l}\right]$ and carries a structure closely related to the first term in Equation (67).

The evolving dynamical equation of ${\ddot{y}}^{l}$ derived in [6] results in

(68) $$\begin{array}{c} {\ddot{y}}^{l}=\frac{-1}{m^{2}}p^{i}M_{ij}^{l}p^{j}+\frac{1}{2m}p^{i}\frac{d}{dt}\left[\frac{i}{\hbar }g_{ij},y^{l}\right]p^{j},\;M_{ij}^{l}:=\frac{1}{2}\frac{\partial g_{ij}\left(x,p\right)}{\partial x_{l}} \end{array}$$

where two of the indices i,j are contracted with momentum, following a behavior such that the underlying geodesic flow, as exhibited by the expectation values, is subjected to the presence of an additional kind of “force” term, which is an essentially quantum effect, and has the consequence of contributing to the forces driving the system [6,19,20,21,22].

This term accounts for the underlying “geometric flow” altering the Hamilton manifold of the underlying geometry [6]. Classically, the sign of the eigenvalues of this term may contribute to the local stability properties of the geodesic flow on the Hamilton manifold, reflecting different behaviors of its geometric evolution [6].

Therefore, our study here on the quantum mechanical Ricci flow is essentially by its nature a geometric flow on a Finsler geometry (a perturbed Ricci flow on a Finsler geometry). The term of Equation (67), in this sense, may affect the stability properties of the perturbed Ricci flow [23,24].

We now turn to the following definitions.

**Definition 4.**  *(Adiabatic Flow)*

*Let $H_{G}$ be a Hilbert space corresponding to a given quantum mechanical system and let ${\hat{H}}_{G}$ be the self-adjoint geometric Hamiltonian generating the evolution of the system as before.*

*Let the Riemann curvature operator $R_{qmn}^{l}$, the Ricci curvature operator $R_{ij}$ and the $\Xi_{ij}$ operator be as before.*

*Define $A^{l}$ to be a bounded operator acting on $H_{G}$, considered to be small, as before.*

*Let the quantum mechanical system at a given time undergo irreversible evolution such that the Hermiticity of the tensor metric operator $g_{ij}\left(x\right)$ is no longer preserved.*

*Then, an adiabatic evolution is defined to be*

(69) $$\Xi_{ij}\approx \frac{m^{2}}{{\left(4\hbar \right)}^{2}}g_{nm}M_{ij}^{m}\left[\frac{\partial g_{nk}}{\partial t},y^{k}\right]$$

*A generalized quantum mechanical adiabatic flow equation reads*

(70) $$\begin{array}{c} \frac{\partial g_{ij}}{\partial t}=-2\lambda \left(R_{ij}-\Xi_{ij}\right),\;\lambda :=\frac{i\hbar }{m}\\ \;where\;\Xi_{ij}:=\frac{m^{2}}{{\left(4\hbar \right)}^{2}}g_{nm}M_{ij}^{m}\left[\frac{\partial g_{nk}}{\partial t},y^{k}\right] \end{array}$$

*so that $R_{ij}$ and $\Xi_{ij}$ are functions of the tensor metric operator $g_{ij}$ acting on $H_{G}$ (to be described below in Equation (75)).*

Classically, following the canonical quantization (Dirac’s correspondence principle), $2\lambda \Xi_{ij}^{classic}$ becomes (${\left\{\cdot ,\cdot \right\}}_{PB}$ is the Poisson bracket)

(71) $$\begin{array}{c} 2\lambda \Xi_{ij}^{classic}=\frac{m}{8}g_{nm}M_{ij}^{m}{\left\{\frac{\partial g_{nk}}{\partial t},y^{k}\right\}}_{PB} \end{array}$$

Then, having defined the adiabatic flow, we define “distance” within the operator space and establish the perturbed Ricci flow as the underlying geometry induced by the operator metric space.

**Definition 5.**  *(Quantum Mechanical Ricci Flow)*

*Let $H_{G}$ be a Hilbert space corresponding to a given quantum mechanical system and let ${\hat{H}}_{G}$ be the self-adjoint geometric Hamiltonian generating the evolution of the system as before.*

*Let the Riemann curvature operator $R_{qmn}^{l}$, the Ricci curvature operator $R_{ij}$ and the $\Xi_{ij}$ operator be as before.*

*Let the adiabatic evolution and the adiabatic flow be as before.*

*Define the operator space χ to be a metric space with a distance function $\left|\right|{\hat{\delta }}_{ij}\left|\right|\left(t\right)$ for each $t\in R^{+}$ such that $\left|\right|{\hat{\delta }}_{ij}\left|\right|$ denotes curves from $\left[t_{s},t_{s}+\epsilon_{s}\right)$ into χ by*

(72) $$\begin{array}{c} \left|\right|{\hat{\delta }}_{ij}||\left(t\right):=\underset{t^{\prime },t^{\prime \prime }\in \left[t_{s},t_{s}+\epsilon_{s}\right)}{\sup}\left|\right|g_{ij}\left(t^{\prime }\right)-g_{ij}\left(t^{\prime \prime }\right){\left|\right|}_{\chi }\\ \mathrm{where}\;h_{ij}:=g_{ij}\left(t^{\prime }\right)-g_{ij}\left(t^{\prime \prime }\right),\;||h_{ij}{\left|\right|}_{\chi }:=\underset{\phi ,\psi \in H_{G}}{\sup}\sqrt{\langle \phi |h_{ij}^{†}h_{ij}|\psi \rangle } \end{array}$$

*for all states $\phi ,\psi \in H_{G}$ in n-dimensional $H_{G}\left(M^{n}\right):=L^{2}\left(M^{n}\right)$, expressed in the Hamilton representation as before. Denote the space of continuous curves for which this norm is finite by $C(\left[t_{0},T\right);\chi )$ for a time interval $\left[t_{0},T\right)$, where $\left|\right|{\hat{\delta }}_{ij}\left|\right|\left(t\right)$ lies in the space $C(\left[t_{0},T\right);\chi )$.*

*Define the trajectory *Φ* corresponding to an initial curve $\left|\right|\hat{\delta }\left|\right|\left(t_{0}\right)$ to be [19,20]*

(73) $$\begin{array}{c} \Phi_{ij}\left(t\right):=\left\{|\right|{\hat{\delta }}_{ij}\left||\left(t\right)\;|\;|\right|{\hat{\delta }}_{ij}\left||\left(t\right),|\right|{\hat{\delta }}_{ij}\left|\right|\left(t_{0}\right)\in C(\left[t_{0},T\right);\chi ){\}}_{s=0,t\in [t_{0},T)}^{\infty } \end{array}$$

*i.e., Φ(t) is the set of $\left[t_{0},T\right)\times \left\{|\right|{\hat{\delta }}_{ij}\left|\right|\left(t\right):t\in \left[t_{0},T\right)\}$ reached in the course of the evolution of the system from an initial $\left|\right|{\hat{\delta }}_{ij}\left|\right|\left(t_{0}\right)$. The trajectory Φ(t) is then said to be the quantum mechanical geometric flow within the $C(\left[t_{0},T\right);\chi )$ space.*

*Given any metric $g_{0}=g_{ij}\left(x\right)$ on n-manifold $\left(M^{n},g_{ij}\left(x\right)\right)$, a generalized quantum mechanical Ricci flow equation within the metric operator space $C(\left[t_{0},T\right);\chi )$ reads*

(74) $$\begin{array}{c} \frac{\partial g_{ij}}{\partial t}=-2\lambda \left(R_{ij}-\Xi_{ij}\right),\;\lambda :=\frac{i\hbar }{m}\\ \;where\;\Xi_{ij}:=\frac{m^{2}}{{\left(4\hbar \right)}^{2}}g_{nm}M_{ij}^{m}\left[\frac{\partial g_{nk}}{\partial t},y^{k}\right],\;R_{ij},\Xi_{ij}\in C(\left[t_{0},T\right);\chi )\\ and\;g_{ij}\left(0\right)=g_{ij}\left(x\right),\;\Xi_{ij}\left(0\right)=\Xi_{ij}\left(g_{ij}\left(x\right)\right),\;R_{ij}\left(0\right)=R_{ij}\left(g_{ij}\left(x\right)\right) \end{array}$$

*so that $R_{ij}$ and $\Xi_{ij}$ are tensors acting on $H_{G}$ and has at least a perturbed unique smooth solution $g_{ij}\left(t\right)$ on M×[0,T), so that $g\left(t\right)\approx \left(1-\epsilon \right)g_{Ric}\left(t\right)$ is a small perturbation of the Ricci flow solution $g_{Ric}\left(t\right)$ (ϵ is positive or negative), and g(t) is a smooth one-parameter family of metrics on the n-manifold $\left(M^{n},g_{ij}\left(t\right)\right)$.*

We now turn to the final step, which suggests a definition for “local instability” in the quantum theory.

This notion relies on the work of Bahuaud et al. [23,24,25], proving that the Ricci flow for complete metrics with bounded geometry depends continuously on initial conditions for a finite time with no loss of regularity [24]. Their work leads to a general theorem (Theorem 3.6. in [24]) about convergence stability around any given converging flow trajectory in any geometric flow, i.e., it applies not only to the Ricci flow but to more general geometric flows as well.

We suggest relating the stability of the quantum mechanical system with the principle of convergence stability for geometric flows in the operator metric space, which is the combination of the continuous dependence of the flow on initial conditions, with the stability of fixed points [23,24].

We first express $\Xi_{ij}$ in the $\left\{y^{l}\right\}$ coordinate base, substituting Equation (48) in Equation (67), expressed in the Hamilton representation

(75) $$\begin{array}{c} \Xi_{ij}\approx \frac{im}{8\hbar }g_{nm}M_{ij}^{m}\left[{\dot{y}}^{k},g_{nk}\right]=\frac{1}{2}g_{nm}M_{ij}^{m}g^{ka}\frac{\partial g_{nk}}{\partial y^{a}}=\frac{1}{2}g_{nm}M_{ij}^{m}M_{nk}^{k} \end{array}$$

such that the generalized *quantum mechanical Ricci flow* equation within the metric operator space $C(\left[t_{0},T\right);\chi )$ reads

(76) $$\begin{array}{c} \frac{\partial g_{ij}}{\partial t}=-2\lambda \left(R_{ij}-\Xi_{ij}\right),\;\lambda :=\frac{i\hbar }{m}\\ \;where\;\Xi_{ij}:=\frac{1}{2}g_{nm}M_{ij}^{m}M_{nk}^{k},\;R_{ij},\Xi_{ij}\in C(\left[t_{0},T\right);\chi )\\ and\;g_{ij}\left(0\right)=g_{ij}\left(x\right),\;\Xi_{ij}\left(0\right)=\Xi_{ij}\left(g_{ij}\left(x\right)\right) \end{array}$$

We identify this geometric flow, under the adiabatic flow constraint, as the perturbed Ricci flow, where the perturbation term acts as an additional geometric correction.

We now invoke the the work of Bahuaud et al. [23,24,25], studying the convergence stability for the Ricci flow on manifolds with bounded geometry and we apply their theorem (3.6.) [24] below.

The Banach spaces on which we let these operators act are little $H\ddot{o}lder$ spaces. We work in the topology of little $H\ddot{o}lder$ spaces of metrics as in [23,24,25].

Suppose that the geometric flow Equation (76) is any ungauged geometric flow which admits a gauging, i.e., the addition of a term G(g) tangent to the diffeomorphism orbit and such that D(F+G) is an elliptic geometric operator which is admissible in the sense of [25] (see discussions in [24,26,27]).

**Theorem 1.**  *(Convergence Stability)*

*Let $H_{G}$ be a Hilbert space corresponding to a given quantum mechanical system and let ${\hat{H}}_{G}$ be the self-adjoint geometric Hamiltonian generating the evolution of the system as before.*

*Let the quantum mechanical Ricci flow (which is said to be the perturbed Ricci flow) be as before and the geometric flow equation reads*

(77) $$\begin{array}{c} \frac{\partial g_{ij}}{\partial t}=-2\lambda \left(R_{ij}-\Xi_{ij}\right),\;\lambda :=\frac{i\hbar }{m}\\ \;where\;\Xi_{ij}:=\frac{1}{2}g_{nm}M_{ij}^{m}M_{nk}^{k},\;R_{ij},\Xi_{ij}\in C(\left[t_{0},T\right);\chi )\\ and\;g_{ij}\left(0\right)=g_{ij}\left(x\right),\;\Xi_{ij}\left(0\right)=\Xi_{ij}\left(g_{ij}\left(x\right)\right),\;R_{ij}\left(0\right)=R_{ij}\left(g_{ij}\left(x\right)\right) \end{array}$$

*given that $g_{ij}\left(x\right)$ is any metric of a given geometry on n-manifold $\left(M^{n},g_{ij}\left(x\right)\right)$.*

*Let the quantum mechanical Ricci flow be an ungauged geometric flow. If the addition of a term $G_{ij}$ tangent to the diffeomorphism orbit and $D(R_{ij}-\Xi_{ij}+G_{ij})$ is an elliptic geometric operator which is admissible in the sense of [25], the quantum mechanical Ricci flow is said to be admitting a gauging.*

*Let $g_{h}$ be any stationary point of the quantum mechanical Ricci flow equation, both ungauged and gauged flows [23,24,25], which is strictly stable. Let $g_{R}\left(t_{0}\right)$ be any metric which is the initial point of a solution $g_{R}\left(t\right)$ to the quantum mechanical Ricci flow (ungauged flow).*

*Then for any metric g sufficiently near to $g_{R}$ in an appropriate weighted $H\ddot{o}lder$ norm $g-g_{R}\in C_{\mu }^{k,\alpha }\left(M\right)$ for some k≥2 and μ∈(0,n−1), the ungauged flow g(t) converges to $g_{h}$ and the entire trajectory g(t) remains close to $g_{R}\left(t\right)$ for all t. The quantum mechanical system is then said to be stable in $H_{G}$ along the evolution.*

*However, if at some time $t^{\prime }:g\left(t^{\prime }\right)-g_{R}\left(t^{\prime }\right)\notin C_{\mu }^{k,\alpha }\left(M\right)$, the quantum mechanical system is then said to be locally unstable in $H_{G}$ at $t^{\prime }$ and the entire trajectory g(t) may not remain close to $g_{R}\left(t\right)$ for all t.*

## 3. Conclusions

This work attempts to address the basic notion of quantum mechanical dynamical stability and attempts to provide definitions for stability, instability and local instability within the quantum mechanical evolution.

A new geometric approach is introduced based on the construction of an underlying perturbed Ricci flow within the metric operator space which provides the basis for the notion of convergence stability in the quantum mechanical theory introduced in this work.

The detailed analysis results in a theorem which addresses the quantum mechanical evolution defining instability properties of the quantum mechanical dynamical equations in a structure of geometric flows, valid beyond the stability properties of the counterpart classical systems regardless the Ehrenfest theorem.

Our geometrical formulation of quantum evolution provides a potential framework for introducing a quantum mechanical counterpart to the classical Ricci flow, which has been extensively utilized in various areas of physics. This extension not only broadens the applicability of the Ricci flow to the quantum regime but also offers a sensitive diagnostic tool for advanced systems, such as nanoelectromechanical systems (NEMS). 

## Data Availability

The original contributions presented in this study are included in the article. Further inquiries can be directed to the corresponding author(s).
